# Acting on the Evidence: The Challenges Facing Policy and Practice: Comment on "Barriers and Opportunities for WHO ‘Best Buys’ Non-communicable Disease Policy Adoption and Implementation From a Political Economy Perspective: A Complexity Systematic Review"

**DOI:** 10.34172/ijhpm.9022

**Published:** 2025-05-27

**Authors:** David J. Hunter

**Affiliations:** Newcastle University, Newcastle, UK.

**Keywords:** Public Health, Neoliberalism, Political Economy Approach, Complex Systems Thinking, Implementation Research, Trade and Health

## Abstract

Even in situations where there exists robust evidence on what works and what needs to change to tackle deep-seated and persistent public health challenges, the lack of sustained progress across polities globally remains a cause for concern. Adopting a political economy perspective to better understand why the adoption and implementation of policies to tackle non-communicable diseases (NCDs) continues to be deficient, Loffreda and colleagues’ systematic review of facilitating and inhibiting factors sheds valuable light on the subject. The adoption of a political economy approach is long overdue since it gets to the nub of identifying enablers and barriers to change and how to tackle the latter while strengthening the former. However, whether such an approach will be welcomed by policy-makers or be rejected merits further exploration if research is going to stand any chance of being heeded and acted upon.

 The World Health Organization (WHO) reports that the commercial determinants of non-communicable diseases (NCDs) are responsible for 90% of deaths in the European Region with almost two-thirds of these directly attributed to risk factors arising from four major commercial products—alcohol, tobacco, processed food and drinks, and fossil fuels.^1^ The Region is falling behind in reaching the global NCD target of reducing premature mortality by a third by 2025. The report reaches a damning conclusion: countries “are not implementing even half of the WHO ‘best buy’ policies which have consistently shown to be beneficial” and for which there is robust evidence regarding what works. As the findings from Loffreda and colleagues’ complexity systematic review on the barriers and opportunities of WHO’s “best buys” to tackle NCDs demonstrate, such a conclusion applies globally.^2^ Taking obesity as an example, its prevalence is increasing in every region of the world and, according to the *Lancet*, is the fastest-growing risk factor for disease worldwide. Yet, despite the “reams of policy recommendations designed to reverse rising obesity rates…not one has led to successful and sustained change.”^3^

 Against this bleak backdrop, the systematic review conducted by Loffreda and colleagues is timely. The political economy perspective adopted to examine the role of actors and the manner in which they operate is especially important since if we already have sufficient evidence on both the nature of the risk factors underlying NCDs and on the “best buys” recommended for adoption and implementation, then we need to understand better what is preventing action from happening. This centres on what the authors refer to as the “policy disconnect” between the risk factors associated with NCDs and the policy responses to them.

 While the systematic review does not advance significant new knowledge on the barriers to, and opportunities for, change, it does provide a useful and rigorous synthesis of the available evidence from studies using a political economy lens. It endorses a complex systems approach to better understand what is happening to hamper or facilitate policy take-up and summarises the main barriers and facilitators either preventing the adoption of policy or enabling it. However, the authors’ claim that they “have introduced a new way to look at NCD policies by adopting a complexity perspective and using system thinking” is not fully justified or borne out by the evidence. A WHO report published in 2022 set out a detailed case for adopting a systems thinking approach in the prevention of NCDs.^4^ And an earlier WHO report, published in 2009 in conjunction with the Alliance for Health Policy and Systems Research, mapped out a set of strategies and activities to harness systems thinking to strengthen health systems.^5^ Directing its message to researchers, among others, the report’s aim was “to promote systems thinking as the norm in the design and evaluation of interventions in health systems.” It is a message that researchers have heard.^6^

 Loffreda and colleagues assert that although we know which policies and interventions work and merit implementation, there remains some ignorance over how best to implement them. They argue for implementation research to understand better what policies should be adopted and to ensure they are successfully implemented. Further exploration of the drivers that influence political decisions is called for beyond simply stating that “political will” is lacking. It is a call echoed by others.^7^

 These conclusions are possibly the least convincing part of the systematic review – not because they are not important, which they are, but because they are not especially novel. Nor have they gone unaddressed. There already exists a sizeable literature on policy and/or implementation failure and its causes as well as on those factors that can enable or hasten progress. Some of this literature draws on political economy, or political science, insights and thinking, a key strength of the systematic review. For instance, mention is made of neoliberalism’s negative impact on political decisions and the need for more exploration of the issue. But analyses of neoliberalism already exist – how much further exploration is needed? For example, in an important paper published in 2012, the health economist, Gavin Mooney, examines the power structure and vested interests that permeate health systems and policy around the social determinants of health.^8^ Also adopting a political economy perspective, he concludes that there has been no attempt to address the issue of neoliberalism and the health problems which this form of political economy has created. Hence his call for a new political economy of public health.

 Calling for more research is the common default position among many academics and while there is always a legitimate case to be made for further inquiry, there is also a need to acknowledge when we have sufficient or good enough evidence on a particular topic to justify taking action. Far from yet more research inquiry being needed into the impact of neoliberalism on health, a crucial gap that needs addressing further is deeper analysis of how to confront it and what should replace it. Mooney advocates viewing health care more as a social institution than a commodity, moving from individualism to communitarianism. It is a theme taken up by Littlejohns and colleagues who argue that what such thinking requires is an explicit rejection of neoliberalism, or libertarianism, a form of what the authors call “vulgar individualism.”^9^ This asserts that it is not for the state to promote the health of individuals which is a matter of personal responsibility. In place of such thinking, the authors set out a manifesto for a new social contract incorporating the principles of what they call “social individualism.” It entails using the instruments of government to create the conditions for individual choice and fulfilment. At the heart of social individualism is a focus on prevention and precaution as well as a commitment to social solidarity in the face of widening health inequalities.

 These are also themes which the WHO report on commercial determinants of NCD, cited earlier, addresses. It argues that the power of people’s voice matters and that citizens and civil society can act to reduce the commercial determinants of health (CDHs) and tackle NCDs. In particular, by shifting the public health narrative away from focusing solely on individual responsibility in NCD prevention, citizens and civil society organisations can “help build coalitions, represent affected populations, serve as watchdogs for accountability, can shape policies, and empower communities.” Moreover, such groupings can advocate for change and hold governments and commercial actors to account for “a political economic system” that is able to promote good health.

 There are alternatives to the neoliberal order if governments choose to pursue them. Other economic models are possible which would privilege the health and wider social impacts of policy beyond the pursuit of growth as an end in itself. Too often the fixation on growth and economic liberalization reinforces political and economic systems that prioritise commercial interests over population health and well-being. In the pursuit of public health, addressing the political economic system and rethinking capitalism cannot be ignored.

 Where further research and analysis may be in order is to explore why challenges to the neoliberal order are all too readily ignored, rubbished, dismissed or buried. Is it because they raise uncomfortable truths for policy-makers which they would prefer not to have to confront for fear of having their true beliefs challenged or being held to account for their actions? Loffreda and colleagues do not directly confront this issue which, arguably, is the elephant in the room.

 It has never been easy to conduct research into sensitive policy issues but this applies especially to research of an ethnographic nature designed to illuminate and capture a range of implementation effects beyond the reach of other approaches.^10^ Inconvenient research findings, especially if they reveal conflict and competing perspectives, are unlikely to be welcomed by politicians or policy-makers who have invested heavily in a particular policy and attached to it high symbolic significance in terms of the prevailing dominant political ideology. Hence the preference among policy-makers and politicians for narrow, “scientific” studies, economic analyses or pure “experience.”

 In such a climate, pursuing the type of research advocated by Loffreda and colleagues may not prove straightforward. Even if more implementation studies, particularly those adopting a political economy lens, get funded, questions remain over their impact (or lack of one) if they raise uncomfortable truths. As has been argued by lllsley, among others, “research has little power and is more frequently ignored than adopted.”^11^ For many in the academic community such concerns may appear irrelevant or not their business so long as the research funds continue to flow and peer reviewed publications continue to strengthen academic careers. They may be inclined to pay scant regard to the needs of policy-makers and how dissemination of research might best be undertaken to meet their needs.

 Although understandable, such a view is not held by all those undertaking research into policy who want to see research findings taken up to inform and improve policy responses to problems. Mobilising knowledge in complex systems like health to bring about change should be a matter of concern to researchers motivated to contribute to the science of knowedge-to-action.^12^ While such matters go beyond the scope of Loffreda and colleagues’ systematic review, they are critical to confronting the issues raised in the discussion of the global literature on barriers and opportunities of NCD “best buys.” For example, as is rightly pointed out, trade deals (and their rules) represent a key challenge for governments. The need is to ensure that such deals support rather than hinder efforts to tackle NCDs. And yet, in practice trade deals do not pay much heed to health concerns, especially for governments keen to stimulate economic growth at all costs. Indeed, as has been argued, “the opacity of trade negotiations” and “relative exclusion of health from debates creates a potentially dangerous imbalance.”^13^ In principle, there is no reason why health should not be included in trade negotiations but the fact it is not suggests issues of a more political nature are at play. Unless these are addressed by acknowledging that effective prevention requires regulation of commercial practice related to the harmful use of products, then halting the rise of NCDs will become all but impossible. This is the key message to emerge from a *Lancet* Series on the CDHs.^14^

 As the WHO report on the commercial determinants of NCD makes clear, there are significant implications here for the public health community and its competencies. Capacity-building and continuous education are essential to enable public health actors to understand issues arising from trade and health and to equip them with the knowledge and skills to ensure that health considerations are prioritized in trade agreements. This presents a challenge to the public health community which lacks a proper understanding of the impact of potential constraints that trade may impose on the public’s health and how these might be mitigated. Policies designed to improve the public’s health have focused on individual behaviour change. While helpful, they fall short of the type of action needed and which only governments can undertake. But their failure to take decisive action, despite the evidence testifying to its positive impact, may well reflect the corrosive influence of powerful corporate lobbying on the part of the food and drinks industry. There may be important lessons to learn from actions taken to control smoking in public places. There is also the example of minimum unit pricing for alcohol which was introduced in Scotland despite the lobbying efforts of the drinks industry. The policy was subsequently adopted by Wales and the Republic of Ireland while England remains an outlier. What may be needed is a framework for alcohol control equivalent to the Framework Convention on Tobacco Control.

 Such a discussion reinforces the challenge facing researchers. In addition to closing any gaps in our knowledge through further research, greater attention than has been accorded hitherto should be given to why what is already known about the failure to implement WHO’s “best buys” to tackle NCD, and address the issues raised by the CDHs, is not heeded by policy-makers. Following Illsley and others, this may require researchers finding novel ways to disseminate their work to gain traction over the implementation of research findings and to influence the policy debates surrounding obesity, alcohol misuse, widening health inequalities and so on. As has been noted, academics should not see themselves “as mere observers or students of government [but] must seek to engage” through, for example, providing advice and taking secondments in governments thereby making the borders between academia and government more porous.^15^ Embedding researchers in policy and practice settings is another way forward.^16,17^ A co-production of knowledge approach in which researchers and research users work together to co-create, refine, implement, and evaluate the impact of new knowledge may also be helpful.^18^ In particular, it may mean widening the discourse of research evidence and its impact so that the wider public is engaged and better informed to advocate for change. WHO’s proposal for civil society organisations mentioned earlier deserves support from, and engagement by, academic researchers. Such an approach is in keeping with Loffreda and colleagues’ mention of an advocacy coalition, comprising researchers, civil society health officials and others, to facilitate implementation.

 Unless an effort is made along these lines to close the know-do gap, it is hard to be confident about what, if anything, might change in practice. Under such circumstances, a more likely scenario is that WHO’s “best buys” remain on the shelf.

## Ethical issues

 Not applicable.

## Conflicts of interest

 Author declares that he has no conflicts of interest.

## References

[1] World Health Organization (WHO). Commercial Determinants of Noncommunicable Diseases in the WHO European Region. Copenhagen: WHO; 2024.

[2] Loffreda G, Arakelyan S, Bou-Orm I (2024). Barriers and opportunities for WHO “best buys” non-communicable disease policy adoption and implementation from a political economy perspective: a complexity systematic review. Int J Health Policy Manag.

[3] Horton R (2025). Offline: making obesity matter. Lancet.

[4] World Health Organization (WHO). Systems Thinking for Noncommunicable Disease Prevention Policy: Guidance to Bring Systems Approaches into Practice. Copenhagen: WHO; 2022.

[5] de Savigny D, Adam T. Systems Thinking for Health Systems Strengthening. Geneva: Alliance for Health Policy and Systems Research, WHO; 2009.

[6] Best A, Holmes B (2010). Systems thinking, knowledge and action: towards better models and methods. Evid Policy.

[7] Hunter DJ (2015). Role of politics in understanding complex, messy health systems: an essay by David J Hunter. BMJ.

[8] Mooney G (2012). Neoliberalism is bad for our health. Int J Health Serv.

[9] Littlejohns P, Hunter DJ, Weale A, Johnson J, Khatun T. Making Health Public: A Manifesto for a New Social Contract. Bristol: Policy Press; 2024.

[10] Pollitt C, Harrison S, Hunter DJ, Marnoch G (1990). No hiding place: on the discomforts of researching the contemporary policy process. J Soc Policy.

[11] Illsley R. Professional or Public Health? Sociology in Health and Medicine. The Rock Carling Fellowship. London: Nuffield Provincial Hospitals Trust; 1980.

[12] Holmes BJ, Best A, Davies H (2017). Mobilising knowledge in complex health systems: a call to action. Evid Policy.

[13] Smith R, Hunter DJ, Kingston P, Lodge H. Between COVID-19, Brexit, and New Trade Deals, A Non-Communicable Disease Storm is Brewing. BMJ; 2020. https://blogs.bmj.com/bmj/2020/11/23/between-covid-19-brexit-and-new-trade-deals-a-non-communicable-disease-storm-is-brewing/.

[14] The Lancet (2023). Unravelling the commercial determinants of health. Lancet.

[15] Sanders M (2025). Debate: what can academia contribute to public administration?. Public Money Manag.

[16] Hunter DJ (2019). Meeting the challenge of the “know-do” gap: Comment on “CIHR health system impact fellows: reflections on ‘driving change’ within the health system”. Int J Health Policy Manag.

[17] Cheetham M, Redgate S, van der Graaf P, Humble C, Hunter D, Adamson A (2023). ‘What I really want is academics who want to partner and who care about the outcome’: findings from a mixed-methods study of evidence use in local government in England. Evid Policy.

[18] Kitson A, Powell K, Hoon E, Newbury J, Wilson A, Beilby J (2013). Knowledge translation within a population health study: how do you do it?. Implement Sci.

